# Effects of sex chromosome dosage on corpus callosum morphology in supernumerary sex chromosome aneuploidies

**DOI:** 10.1186/s13293-014-0016-4

**Published:** 2014-10-16

**Authors:** Benjamin S C Wade, Shantanu H Joshi, Martin Reuter, Jonathan D Blumenthal, Arthur W Toga, Paul M Thompson, Jay N Giedd

**Affiliations:** 1 Imaging Genetics Center, Institute for Neuro Imaging and Informatics, USC, 4676 Admiralty Way, Marina del Rey, Los Angeles 90292, CA, USA; 2 Department of Neurology, Ahmanson-Lovelace Brain Mapping Center, UCLA, Los Angeles 90095, CA, USA; 3 Martinos Center for Biomedical Imaging, Massachusetts General Hospital, Charlestown 02129, MA, USA; 4 Institute for Neuro Imaging and Informatics, Keck School of Medicine, USC, Los Angeles 90032, CA, USA; 5 Child Psychiatry Branch, National Institute of Mental Health, Bethesda 20892-1600, MD, USA

**Keywords:** Corpus callosum, Sex chromosomes, Aneuploidies, Statistical shape analysis, Sexual differentiation

## Abstract

**Background:**

Supernumerary sex chromosome aneuploidies (sSCA) are characterized by the presence of one or more additional sex chromosomes in an individual’s karyotype; they affect around 1 in 400 individuals. Although there is high variability, each sSCA subtype has a characteristic set of cognitive and physical phenotypes. Here, we investigated the differences in the morphometry of the human corpus callosum (CC) between sex-matched controls 46,XY (*N* =99), 46,XX (*N* =93), and six unique sSCA karyotypes: 47,XYY (*N* =29), 47,XXY (*N* =58), 48,XXYY (*N* =20), 47,XXX (*N* =30), 48,XXXY (*N* =5), and 49,XXXXY (*N* =6).

**Methods:**

We investigated CC morphometry using local and global area, local curvature of the CC boundary, and between-landmark distance analysis (BLDA). We hypothesized that CC morphometry would vary differentially along a proposed spectrum of Y:X chromosome ratio with supernumerary Y karyotypes having the largest CC areas and supernumerary X karyotypes having significantly smaller CC areas. To investigate this, we defined an sSCA spectrum based on a descending Y:X karyotype ratio: 47,XYY, 46,XY, 48,XXYY, 47,XXY, 48,XXXY, 49,XXXXY, 46,XX, 47,XXX. We similarly explored the effects of both X and Y chromosome numbers within sex. Results of shape-based metrics were analyzed using permutation tests consisting of 5,000 iterations.

**Results:**

Several subregional areas, local curvature, and BLDs differed between groups.

Moderate associations were found between area and curvature in relation to the spectrum and X and Y chromosome counts. BLD was strongly associated with X chromosome count in both male and female groups.

**Conclusions:**

Our results suggest that X- and Y-linked genes have differential effects on CC morphometry. To our knowledge, this is the first study to compare CC morphometry across these extremely rare groups.

## 1 Background

As a result of nondisjunction^a^ during meiosis, around 1:400 individuals are born with supernumerary sex chromosomes aneuploidies (sSCA) [1]. Viable sSCA karyotypes include 47,XYY, 47,XXY, 48,XXYY, 48,XXXY, 49,XXXXY, and 47,XXX^b^. sSCA prevalence decreases exponentially as karyotype count increases. Historically, aneuploidies of the X chromosome have received a great deal of interest due to the large number of X-linked genes expressed in the brain [1],[2] which are strongly associated with cognitive disorders [3].

In typically developing human females, a process of X inactivation occurs to prevent overexpression of X-linked genes; this silences genes from one of the two X chromosomes [4]. Similarly, in the presence of supernumerary X chromosomes, all but one X chromosome is silenced [5]–[8]. Around 15% of the genes in the silenced chromosome remain active [9]; they may contribute to sexual dimorphism. However, in sSCA, the accumulation of unsilenced genes from supernumerary chromosomes and aberrant inactivation patterns may contribute to the characteristic deficits presented in sSCA subtypes.

Physical and cognitive phenotypic variation among sSCA subtypes is high due to complex interactions of chromosome dosage, mosaicism^c^, and sex hormone abnormalities. However, certain phenotypic characteristics are commonly reported. Mean IQ is 0.5–1 standard deviations lower for every additional chromosome present in the subject’s karyotype [1]. Interestingly, subjects with a single supernumerary chromosome (i.e., 47,XXY, 47,XXX, or 47,XYY) usually test within the average IQ range but significantly lower than a typically developing sibling. Verbal IQ is severely affected while performance IQ is relatively spared [10],[11].

Magnetic resonance imaging (MRI) studies of individuals with X-variant sSCA (*sSCA*_*X*_) tend to reveal lower total brain volumes (TBV) and higher ventricular volumes [12]–[14]. Diffusion tensor imaging studies have also reported lower fractional anisotropy in 47,XXY [15]. People with Y-variant sSCA (*sSCA*_*Y*_) show an apparently opposite effect: higher TBV [16]. White matter (WM) hyperintensities^d^ have been reported for both *sSCA*_*Y*_ and *sSCA*_*X*_[14]. Taken together, these findings suggest a chromosomally driven, dosage-dependent spectrum of brain morphometry. Table 1 summarizes sSCA phenotypes and their effects on MRI measures.

**Table 1:** SCA phenotypes

**Karyotype**	**MRI measure**	**Somatic phenotype**	**Cognitive phenotype**
47,XYY	Increased GM/WM volume [57]	Increased height	Decreased IQ [14]
	Decreased insular and frontotemporal volume [57]	Increased head circumference [1]	Antisocial traits [14]
48,XXYY	Enlarged ventricles	Hypogonadism	Developmental delays
	Diffuse WM abnormalities [21]	Tall stature	Learning disability
		Mild craniofacial dysmorphia [51]	Decreased IQ [21]
47,XXY	Decreased TBV [14]	Hypogonadism	Decreased IQ
	Increased ventricular volume [13]	Tall stature	Higher incidence of schizoid personality traits [1]
	WM hyperintensities [14]	Testicular scarring	
	Decreased fractional anisotropy [15]	Low testosterone [1]	
	Decreased GM volume [52]		
48,XXXY	Decreased WM/GM [1]	Tall stature	Decreased IQ
		Decreased testicular volume	Irritability
		Facial dysmorphism [51]	Passivity [20]
49,XXXXY	Decreased TBV	Decreased height [51]	Severe developmental delays [22]
	WM lesions [22]	Mild craniofacial abnormalities [22]	
47,XXX	Decreased TBV [14]	Increased height [1]	Anxiety and impulsivity [22]
		Radio-ulnar synostosis	Decreased IQ [14]

Here, we investigate the morphological variation of the corpus callosum (CC): (1) between the various sSCA subtypes, (2) as a function of Y:X chromosome ratio, and (3) as a function of X and Y chromosome number within sex. We investigate the CC as it is the most prominent WM bundle in the brain, responsible for communication between homologous brain regions. WM projections through the CC to distinct cortical regions are topographically organized, and local abnormalities in CC morphometry may reflect abnormalities in cortical development [17]–[19].

To investigate these aims, we used two approaches: area and shape analysis. Most studies report on area effects, but shape-based methods offer an added dimension of description.

Studies of CC morphometry in sSCA karyotypes are sparse and often limited to case studies. However, previous studies of sSCA cohorts have indicated varying degrees of morphological abnormalities of the CC in these disorders. In a study of 42 47,XXY subjects, Giedd et al. [20] identified no changes in the cross-sectional area of the CC. An ultrasound case study of a 47,XYY fetus revealed agenesis of the CC. In a multi-center study of 95 48,XXYY subjects (35 of which received an MRI scan), Tartaglia et al. [21] identified a wide array of WM abnormalities including agenesis of the CC in two subjects and CC lipomas in three others. Several studies have identified abnormalities of the CC in the 49,XXXXY karyotype. Blumenthal et al. [22] identified thinning of the CC along with various other WM lesions in a cohort of 14 49,XXXXY subjects relative to 42 46,XY controls. In a case study of a 3-year-old male with 49,XXXXY syndrome, Haeusler [23] identified enlarged ventricular volumes and hypoplasia of the subject’s CC. To our knowledge, this is the first study to investigate the shape-based morphological differences of the CC for these exceedingly rare karyotypes.

## 2 Methods

### 2.1 Subjects

Our test subjects consisted of individuals with the following karyotypes: 47,XYY (*N* =29), 47,XXY (*N* =58), 48,XXYY (*N* =20), 48,XXXY (*N* =5), 49,XXXXY (*N* =6), and 47,XXX (*N* =30). All subjects were gonadally male with exception of the 47,XXX karyotype who were all gonadally female. We define sex based on the subjects’ gonadal statuses as the sex chromosomes of our subjects vary between groups. Only non-mosaic subjects were included. Mosaicism status was confirmed with karyotype testing on all subjects. High-resolution G-band karyotyping was performed on phytohemagglutinin-stimulated patient peripheral blood cultures. A minimum of 50 metaphases were analyzed and 3 karyotypes per patient were produced (all karyotyping was performed by Quest Diagnostics or the Cytogenetics Laboratory, Department of Obstetrics and Gynecology, Georgetown University Hospital). Several subjects were undergoing hormonal therapy while others had either previously undergone therapy or never undergone therapy. Hormonal therapy status was not factored into our analyses. Table 2 shows demographic and clinical details for the participants. The control group consisted of 46,XY males (*N* =99) and 46,XX female (*N* =93). Subjects were matched for age, handedness and socioeconomic status (SES).

**Table 2:** Demographic and MRI measures

	**47,XYY**		**Typically developing controls (TDC)**		**48,XXYY**		**47,XXY**		**48,XXXY**		**49,XXXXY**		**47,XXX**	
	***N*** **=29**		***N*** **=192**		***N*** **=20**		***N*** **=58**		***N*** **=5**		***N*** **=6**		***N*** **=30**	
	**Mean**	**SD**	**Mean**	**SD**	**Mean**	**SD**	**Mean**	**SD**	**Mean**	**SD**	**Mean**	**SD**	**Mean**	**SD**
Demographic measures														
Age	12.58	4.96	12.65	5.04	14.23	5.28	12.62	4.98	10.16	7.24	13.28	4.43	12.18	5.51
Gender (M/F)	29/0		99/93		20/0		58/0		5/0		6/0		0/30	
Socioeconomic status (SES)	56.75^a^	22.14	48.41	18.20	46.40	22.54	54.01	21.50	54.80	20.94	64.66^a^	18.08	41.41	17.13
Handedness (R/L/M)	22/4/3		164/12/16		18/1/1		45/6/7		4/0/1		6/0/0		24/2/4	
Full-scale IQ (FSIQ)	90.58^a^	15.41	114.50	12.56	86.52^a^	12.71	96.78^a^	16.65	77.33^a^	4.72	55.50^a^	5.91	95.30^a^	14.42
Verbal IQ (VIQ)	87.58^a^	15.12	114.14	14.23	80.31^a^	12.32	94.70^a^	16.24	73.33^a^	5.85	59.25^a^	8.50	95.20^a^	14.52
Performance-IQ (PIQ)	95.62^a^	17.01	111.88	11.59	95.94^a^	12.43	98.96^a^	16.99	86.66^a^	7.02	57.25^a^	3.30	96.16^a^	15.00
Testosterone replacement therapy (TRT; currently on/ever on/none)	0/0/0		0/0/192		7/7/13		19/23/35		1/2/3		3/4/2		0/0/0	
MRI measures (cc3)														
Intracranial volume (ICV)	1.53e +06^a^	1.31e +05	1.46e +06	1.29e +05	1.43e +06	1.10e +05	1.42e +06	1.42e +05	1.36e +06	4.53e +04	1.24e +06^a^	1.03e +05	1.29e +06^a^	1.25e +05
Gray matter volume (GM)	8.81e +05^a^	8.69e +04	8.43e +05	8.67e +04	8.17e +05	8.24e +04	8.14e +05^a^	6.74e +04	8.08e +05	5.83e +04	7.21e +05^a^	7.98e +04	7.55e +05^a^	8.77e +04
White matter volume (WM)	5.18e +05^a^	6.27e +04	4.89e +05	5.91e +04	4.91e +05	5.82e +04	4.75e +05	7.52e +04	4.46e +05	3.51e +04	3.99e +05^a^	1.23e +04	4.20e +05^a^	4.20e +05^a^

^a^Significant group difference between TDC and diagnostic group.

Written consent was obtained from the adult participants and verbal or written assent from the child participants. Where relevant, written consent from the parents was obtained for participation in this study. The study protocol was approved by the National Institute of Mental Health Institutional Review Board.

### 2.2 Scanning parameters

All images were acquired on a General Electric 1.5-T Signa scanner (General Electric Medical Systems, Waukesha, WI, USA), located at the NIH Clinical Center in Bethesda, Maryland. A sagittal T1-weighted spin-echo sequence was acquired with 5 mm thickness and 1.5-mm gap (FOV =300 mm, acquisition matrix 256 × 128, TR =400 ms, TE =14 ms). A three-dimensional spoiled gradient-recalled echo sequence in the steady-state sequence was used to acquire 124 contiguous 1.5-mm thick slices in the axial plane (TE =5 ms; TR =24 ms; flip angle, 45°, acquisition matrix =256 × 192; number of excitations, 1; field of view, 240 mm; acquisition time, 9 min, 52 s). Only images with minimal or no motion artifact were accepted for the study.

### 2.3 Corpus callosum tracing procedure

All CC images were acquired from manual tracings of the raw images. Manual tracing of the CC was performed by a single rater, BW, with a high intra-rater reliability (ICC >0.95 for repeated area measures). Tracing was performed in the mid-sagittal slice of the image using the MIPAV software (Medical Image Processing, Analysis and Visualization version 4.3.1 http://mipav.cit.nih.gov/). Prior to tracing, all images were aligned to a standard orientation using methods previously reported in [24]. To extract a binary representation of the CC, an elliptical region of interest (ROI) tightly bounding the CC was thresholded such that image intensities consistent with WM were set to a value of 1 (white) while all other intensities were set to 0 (black). Within this ROI, the rater manually removed all non-CC WM such as WM associated with the *lamina terminalis* and fornix.

### 2.4 Parametric boundary

**Figure 1:**
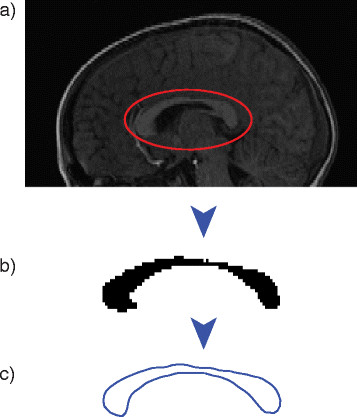
**Extraction and parameterization of corpus callosum boundary.** Illustration of the **(a)** raw SPGR image, **(b)** extracted binary callosum, and **(c)** parametric boundary of callosum as represented in MATLAB.

To obtain the boundary of a given CC, its binary representation was read into MATLAB®. Using the Image Processing Toolbox, the function *bwboundaries* was used to obtain the x-y coordinates of the CC boundary. The elements of each *x*- and *y*-component vector, **u** and **v**, were first downsampled to 100 elements each and subsequently low-pass filtered with a Gaussian filter (height =6, sigma =3) to smooth the boundaries of the CCs. We corrected for primary axis tilt by setting the vertical coordinates of the most anterior point in the *genu* to equal the posterior most point of the splenium. Finally, the parameterized boundaries were translated to place their centroid at the origin to ensure spatial normalization. Figure 1 illustrates the procedure for extracting the parameterized callosal boundary. For each subject, this callosal boundary was used as the structural shape representation of the CC and was analyzed using the morphometric methods described below.

### 2.5 Callosal morphometry

We examined the effects of dosage on different morphological predictors derived from the boundary. Prior studies have performed CC morphometry using well-established methods that have studied boundary thickness [25] or areas of regional parcellations [26]–[29]. Thickness and area depend on the local width and global size of the callosal boundary and are related to the physical dimensions of the CC. Different from the size or area approach is the shape-based approach that analyzes only the geometric information present in the boundary by removing the confounding variables such as scale and pose (location and orientation).

For the thickness and area-based approaches, brain volume and orientation are standardized by first registering the T1-weighted structural images to each other or to an atlas and then tracing the callosal curves. Alternately, the transformation from the registration process can be applied to the natively traced callosal boundaries. This extra step of registration is not needed for shape-based analysis.

In our work, to account for the variability in brain size, orientation, and pose, we registered the shapes of the natively traced callosal curves using a shape-space approach [30]. This approach represents shapes of curves as elements of an infinite dimensional nonlinear space and achieves an elastic correspondence using a Riemannian metric that is fully invariant to reparameterizations of curves. The process of finding the correspondence between two shapes involves finding a shortest path or a geodesic on the shape space. By construction, this geodesic is invariant to translation (pose), rotation (orientation), scale (size), and reparameterization (variability of the speed of tracing of the curve). For our analysis, we investigated size- and shape-based measures to study low- and high-level features of the CC and how they are associated with karyotype. These features were extracted and defined using the parameterized callosal boundaries. Specifically, we analyzed area, boundary curvature, and pairwise landmark point relationships using between-landmark distance analysis (BLDA). The latter two metrics, curvature and BLDA, describe local shape deformations.

**Figure 2:**
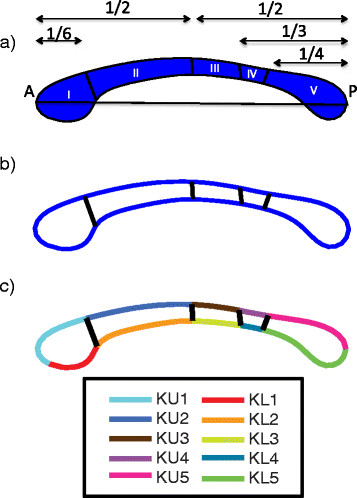
**Hofer-Frahm divisions and curvature boundary segments.** A depiction of the **(a)** Hofer-Frahm subdivisions of the callosum based on diffusion image white matter tractography, **(b)** the representation of these divisions on the parametric boundary in MATLAB, and **(c)** the ten segments of the callosal boundary used to measure average local curvature.

Using diffusion tensor imaging and fiber tractography, Hofer [27] identified five anatomically separable regions of the callosum based on the traversing WM’s cortical termination. They isolated vertically directed segments of the CC that were intended to represent fibers projecting to frontal, motor, sensory, as well as parietal, temporal, and occipital areas. Figure 2a shows the Hofer-Frahm subdivisions corresponding to the following WM projections: (I) prefrontal, (II) premotor and supplementary motor cortices, (III) motor, (IV) sensory, (V) parietal, occipital, and temporal cortices, and the corresponding lengths going from the anterior to the posterior landmark [27].

Figure 2b illustrates the representation of the Hofer-Frahm divisions given by our in-house MATLAB scripts. Appropriate coordinates for the four partitioning lines along the boundary were obtained by identifying the nearest *x-y* coordinates of the boundary satisfying the proportion of total CC length to the Hofer-Frahm subregion. For example, the boundary between regions 2 and 3 occurs at ½ of the CC length. So, the coordinates designating this division would be found by identifying the *x* coordinates for the upper and lower boundaries nearest to ½ of CC length, using the most anterior point of the *genu* as the origin. Due to the discretization and downsampling of the boundary, there is often a slight horizontal offset in the correspondence of the upper and lower ends of the boundary. However, this effect is negligible given that a strictly vertical partition of the underlying traversing WM fibers is no more probable than what is given by this artifact.

### 2.6 Callosal area

For calculating the area, we consider the discretized finite set of vertices given in **u** and **v**. Due to its geometry, the discretized CC becomes a non-self-intersecting, closed polygon. The area is then found by traversing all edges of the polygon and by adding the area of the rectangle (enclosed between the top vertex of the edge and the Y axis) to the left of the edge when traversing downward and subtracting the area of the rectangle when traversing upward. A factor of ½ times the width of the edge is added to account for the extra area whenever the edge is not perfectly perpendicular to the X axis. MATLAB’s *polyarea* function implements this algorithm and computes the area enclosed by the callosal boundary. Both the global callosal and the Hofer-Frahm subregional area were calculated from the vertex coordinates in **u** and **v**.

### 2.7 Callosal regional curvature

The curvature of a boundary can be thought of as the degree to which a local segment of the boundary deviates from appearing spatially flat. It encodes the local rate of change of the line tangent to the local boundary. Formally, for a two-dimensional, parametrically defined boundary given by *x* = *x*(*t*) and *y* = *y*(*t*), the extrinsic curvature is defined as,

(1) $$\kappa =\;\frac{x'y''-\;y'x''}{{\left(x^{'2}+\;y^{'2}\right)}^{3/2}}$$

As in the case of area, local curvature was calculated by partitioning the boundary of the CC into the five Hofer-Frahm regions along its superior and inferior bounds, providing a total of ten sections for examination on each CC. We refer to individual boundary segments as curvature upper (*κ*_*u*_) 1–5 and curvature lower (*κ*_*l*_) 1–5. The constituent points within each section were averaged to provide a single metric of comparison. Figure 2c illustrates the partitions used to compute the local curvature.

### 2.8 Between-landmark distance analysis

Instead of using the Procrustes-based [31] landmark alignment, we used a method that analyzes pairwise landmark distances based on the concept of the Euclidean distance matrix analysis (EDMA) [32],[33] for landmark-based morphometry of the CC. EDMA analyzes the localized variation at each landmark compared to the global landmark shape variation assessed by the Procrustes alignment procedure. The Procrustes method compares landmarks across shapes, whereas EDMA first computes a pairwise Euclidean distance matrix (EDM) for all the landmark points for each shape, and then compares the EDMs across multiple shapes. EDMA is invariant to translation and rotation, but like the Procrustes method, it needs to be normalized to ensure invariance to scale. EDMA does define an invariant statistic known as the maximal invariant in the space of EDM landmark configurations, but we followed a different approach here. We calculated the Euclidean distance matrix consisting of the pairwise distances between landmarks (same as EDM) but compared the distance matrices directly across subjects. This is possible as the callosal boundaries are already registered to each other, so one does not need an additional invariant statistic that is computed in EDMA.

For the CC, 14 landmarks were accurately selected using an automated method, programmed in MATLAB. Table 3 lists the landmarks and abbreviations used in this study, which were chosen for their reproducibility across subjects and their ease of programming. The landmarks were based on the intrinsic geometry of the CC. Eight of the landmarks corresponded to the superior and inferior divisions of the Hofer-Frahm partitions and offered insight into the structure’s primary axis thickness. The landmarks anterior genu (AG) and posterior splenium (PS) corresponded simply to the most anterior and most posterior points of the CC, respectively, and were identified by taking the coordinate pairs of the boundary with the minimum and maximum *x* values. Together, AG and PS measured the length of the CC. Posterior genu (PG) and anterior splenium (AS) were located by identifying the nearest neighboring y coordinate to the AG and PS, respectively, on the lower boundary of the CC curve. Inferior genu (IG) and inferior splenium (IS) were then identified by finding the coordinate pairs with the minimum *y* values in the anterior and posterior halves of the CC. Taken together, the AG-IG-PG complex anteriorly and AS-IS-PS complex posteriorly provided a proxy measure for the bulbosities of the *genu* and the *splenium*.

**Table 3:** Landmark abbreviation key

**Acronym**	**Landmark**
AG	Anterior genu
IG	Inferior genu
PG	Posterior genu
PSG	Posterior superior genu
PIG	Posterior inferior genu
ASI	Anterior superior isthmus
AII	Anterior inferior isthmus
PSI	Posterior superior isthmus
PII	Posterior inferior isthmus
ASS	Anterior superior splenium
AIS	Anterior inferior splenium
AS	Anterior splenium
IS	Inferior splenium
PS	Posterior splenium

**Figure 3:**
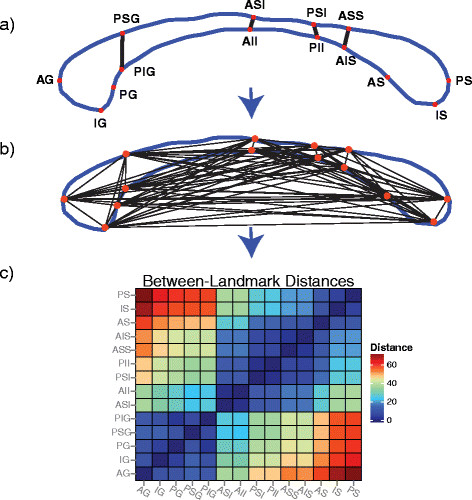
**Between-landmark distance analysis (BLDA) pipeline. (a)** The locations of the 14 landmarks on the parametric boundary of the callosum, **(b)** example trace distances between pairwise landmarks, and **(c)** matrix representation of pairwise distance.

Figure 3 illustrates the location of the landmarks on the boundary of the CC, the pairwise connections between each landmark point, and the computed between-landmark distance matrix (BLDM; 14-by-14 matrix) for the landmarks. Each unique element of the BLDM constitutes a metric for between-group comparisons.

### 2.9 Definition of the karyotype spectrum

As our sample was not restricted to subjects with supernumerary aneuploidies of a single type of sex chromosome but included subjects with supernumerary X and Y karyotypes, we needed to define an ordering for the karyotype spectrum involving both *sSCA*_*Y*_ and *sSCA*_*X*_ karyotypes. Based on prior studies of supernumerary Y chromosomes increasing TBV [16] and the supernumerary X phenotype leading to smaller TBV [12],[14], we ordered our spectrum in descending order of Y:X chromosome ratio in each karyotype, yielding a spectrum of the following order: 47,XYY (2:1), 46,XY (1:1) 47,XXYY (1:1), 47,XXY (1:2), 48,XXXY (1:3), 49,XXXXY (1:4), 46,XX (0:2) 47,XXX (0:3). The relative ordering of 46,XY ahead of 47,XXYY was a special, arbitrary case as the ratio of 46,XY is equal to that of 47,XXYY.

### 2.10 Statistical methods

The Riemannian framework for shape matching of callosal curves [30] can be used to compute invariant statistics such as shape averages and covariances on the tangent space of shapes. The shape average is computed by minimizing the sum squared geodesic distances (geodesic variance) between all the shapes in the population. This shape average is computed intrinsically, i.e., without performing Euclidean averaging, directly on the shape space. A single callosal shape average was computed for the entire population, and all the individual shapes were registered to this average. As the shape average was computed from the population, there was no bias due to choosing a template or an atlas callosal shape for registration.

For each region, we compared the callosal regional areas across all karyotypes. Curvature was calculated on the individual shapes after they were registered to the mean shape. As before, for the group analysis of the between-landmark distance matrices, there was no explicit landmark registration necessary as the landmarks were defined on the registered shapes.

We calculated all $\left(\begin{array}{c} 7\\ 2 \end{array}\right)\;=21$ possible groupwise comparisons for each metric, comparing only male controls to male subjects and female controls to female subjects. Because subjects become exponentially rarer as chromosome count increases, several of the test groups were small enough to merit concern over the validity of implementing standard parametric statistical tests. Instead, we used non-parametric permutation tests of the regression slope for each comparison to avoid violating assumptions of parametric tests. The procedure for this type of permutation test is as follows. (1) The coefficients (slopes) of the regression model’s predictor variables are obtained. (2) Subsequently, the group labels of the model’s predictors are permuted, resampled without replacement, keeping group sizes fixed at their original sizes. (3) Using the resampled group labels, the model’s coefficients are recalculated. (4) This process of resampling repeats for *k* iterations. (5) The significance of the original model is assessed by identifying the proportion of resampled coefficients of the main effect that are more extreme than the original model’s main effect coefficient. For our purposes, each test consisted of 5,000 permutations of the slope using in-house R [34] scripts where the main effect was either, position along Y:X spectrum, number of X-chromosomes or number of Y-chromosomes within sex. For each comparison, we report which results are significant both before and after adjusting for the false discovery rate (FDR).

We investigated the presence of a dosage ratio effect in the metrics by regressing each metric against an enumeration of the karyotype spectrum. Concretely, each subject was assigned a number, 1 through 8, based on their karyotype’s position in the Y:X spectrum where 47,XYY =1 and 47,XXX =8. The coefficient of this Y:X position after correcting for age and ICV reflects the association of the Y:X spectrum with a given metric. Similarly, we regressed metrics against the number of Xs or Ys present in a subject’s karyotype to identify an association with X or Y chromosome count within each sex.

For all tests, we consider an alpha or FDR-adjusted *q* value^e^ of 0.05 to be the threshold for significance. FDR was performed for each family of tests rather than for all tests at once (i.e., we controlled for the number of all pairwise tests of global area, *κ*_*u*,1_, etc., separately).

## 3 Results

**Figure 4:**
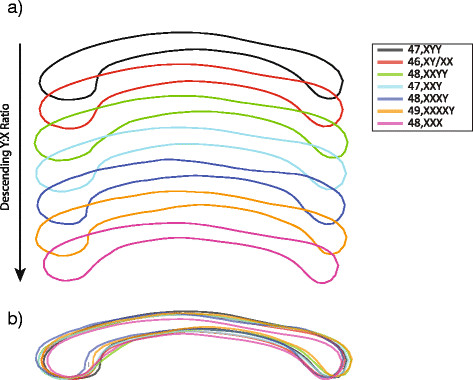
**Average callosum shapes by karyotype. (a)** Average callosum shapes along the karyotype spectrum and **(b)** an overlay of the average shapes.

Figure 4 shows the average callosal shapes associated with each karyotype along the proposed karyotype spectrum. Visual assessment of the shape averages suggests numerous groupwise morphological deviations: (1) from the typical 46,XY/XX group, (2) along the karyotype spectrum, and (3) between groups not adjacent along the spectrum. Most area-based variation along the spectrum appears somewhat linear. However, it is more difficult to determine from visual inspection whether shape-based metrics vary linearly along the spectrum.

### 3.1 Area

**Figure 5:**
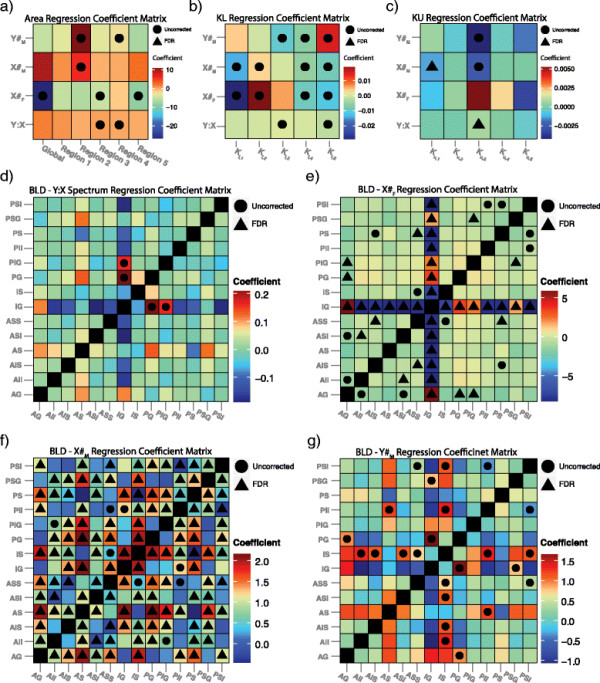
**Summary of chromosome dosage-metric associations.** Coefficient matrices of the associations between the **(a)** area, **(b)** lower boundary curvature, **(c)** upper boundary curvature and **(d-g)** between-landmark distance and the (1) Y:X ratio spectrum, (2) number of X-chromosomes within female karyotypes (*X#*_*F*_), (3) number of X-chromosomes within male karyotypes (*X#*_*M*_), and (4) number of Y-chromosomes within male karyotypes (*Y#*_*M*_). The interpretation of the coefficient in each intersection is simply that for every unit increase in the given spectrum, the metric of interest changes by the amount given by the coefficient (e.g., for every extra X chromosome in the male karyotype, the global area of the callosum decreases by approximately 8 mm^2^). A circle is placed in intersections containing coefficients significant at the 0.05 level prior to correction for multiple comparisons while a triangle is present for coefficients significant after FDR correction.

**Figure 6:**
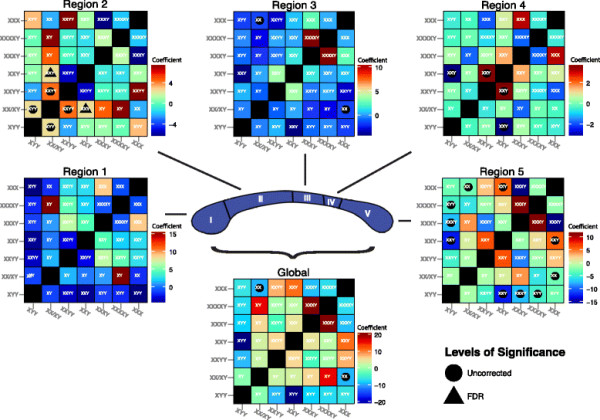
**Global and subregion areas by karyotype.** Boxplots of the global and regional callosal areas associated with each karyotype.

**Figure 7:**
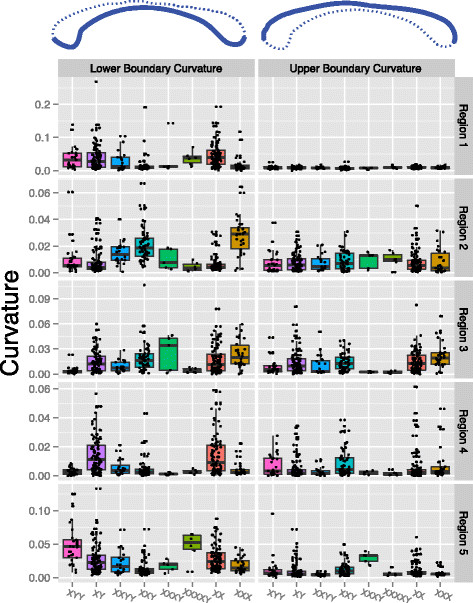
**Groupwise coefficient matrices of area by karyotype.** Groupwise comparisons of area by callosum region. The elements of each matrix contain the coefficient of *Karyotype* resulting from the multiple linear regression model Area_*i*_ = *β*_0_ + *β*_1_ Age_*i*_ + *β*_2_ ICV_*i*_ + *β*_3_ Karyotype_*i*_ + *ϵ*_*i*_. The coefficient is essentially the slope of the least squares line between the areas of the two karyotypes after adjusting for age and ICV. To clarify the direction of the effect, we print the name of the karyotype with the greatest area in the intersection. A circle is placed in intersections containing coefficients significant at the 0.05 level prior to correction for multiple comparisons while a triangle is present for coefficients significant after FDR correction.

Global area was significantly correlated with X chromosome number in females only prior to FDR correction (*P* <0.05; *β*_# *X*_ = −26.67) indicating a significantly lower CC area in 47,XXX females relative to typically developing 46,XX females, see Figure 5a. Several subregions of the CC were weakly associated with the Y:X spectrum or individual Y or X chromosome counts. For instance, region 2 was associated with Y chromosome count in men (*P* <0.05; *β*_# *Y*_ =11.22) suggesting a general increase in this subregion in the presence of supernumerary Y chromosomes. However, none of these associations survive FDR correction and, therefore, must be interpreted with caution. Figure 6 illustrates the pattern of global and subregional CC area by karyotype while Figure 7 reports the coefficients associated with each pairwise comparison of global and subregional areas.

### 3.2 Curvature

**Figure 8:**
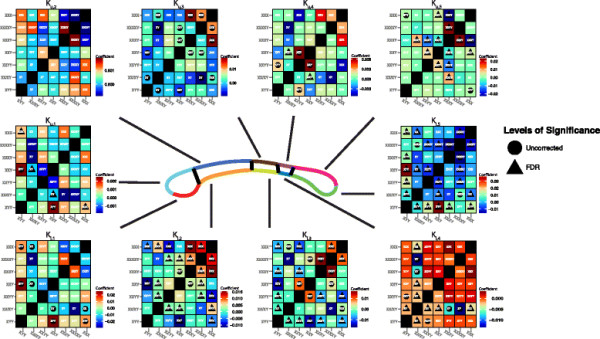
**Average local curvature by karyotype.** Boxplots of average local curvature of the callosal boundary by karyotype.

The boxplots in Figure 8 illustrate the patterns of local curvature by karyotype. After FDR, *κ*_*u*,1_ was significantly associated with the number of X chromosomes in males (*Q* <0.001; *β*_# *X*_ = −0.001). *Κ*_*u*,3_ was associated with the Y:X spectrum (*Q* <0.04; *β*_# *Y* : *X*_ =0.0007). No other regions of the upper boundary curvature survived FDR correction; however, *κ*_*u*,3_was additionally associated with both X and Y count in males only (*P* <0.05; *β*_# *X*_ =0.002 and *P* <0.05; *β*_# *Y*_ =0.004), see Figure 5c. A substantial number of pairwise differences were significant in the upper CC boundary; however, most did not survive FDR. Following FDR, the *κ*_*u*,1_ and *κ*_*u*,4_ segments remained significantly different between 46,XY and 47,XXY males (*Q* <0.05; *β*_*DX*_ = −0.0004 and *Q* <0.05; *β*_*DX*_ =0.0008).

**Figure 9:**
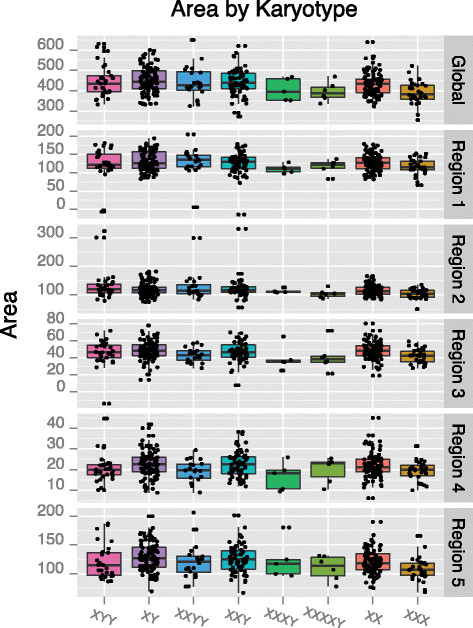
**Groupwise coefficient matrices of average local curvature by karyotype.** Groupwise comparisons of average curvature by callosum region. The elements of each matrix contain the coefficient of *Karyotype* resulting from the multiple linear regression model Curvature_*i*_ = *β*_0_ + *β*_1_ Age_*i*_ + *β*_2_ ICV_*i*_ + *β*_3_ Karyotype_*i*_ + *ϵ*_*i*_. We print the name of the karyotype with the greatest area in the intersection to clarify the direction of the effect. A circle is placed in intersections containing coefficients significant at the 0.05 level prior to correction for multiple comparisons while a triangle is present for coefficients significant after FDR correction.

The curvature of the lower CC boundary exhibited numerous associations with sex-specific chromosome counts and the Y:X spectrum, Figure 5b. However, while numerous, these associations were not robust enough to survive FDR. Despite weak associations with linear karyotype orderings, numerous pairwise differences in lower boundary curvature managed to survive FDR. Only *κ*_*l*,1_ had no robust pairwise differences. Refer to Figure 9 for the groupwise differences in curvature.

The different patterns of curvature differences between upper and lower boundaries may suggest a differential effect of chromosome ratio on the upper and lower boundary of the CC or simply highlight a relatively lower degree of variation in curvature in the upper boundary compared to the lower boundary. Alternatively, these differences may arise due to ventricular expansions in sSCA disorders, which would likely alter the lower boundary of the CC most readily due to its immediate adjacency to the ventricles.

### 3.3 Between-landmark distance analysis

BLDA revealed numerous local shape variations between karyotypes. The distances between the IG and both PG (*P* <0.05; *β*_*Y* : *X*_ =0.21) and posterior inferior genu (PIG) (*P* <0.05; *β*_*Y* : *X*_ =0.16) were significantly associated with the Y:X spectrum prior to FDR but did not manage to survive FDR, see Figure 5d. Among the female-only groups, numerous BLDs were significantly associated with X chromosome count Figure 5e. Most notably, the distance between the IG and every other landmark was robustly associated with X count after FDR. Several other landmark parings remained significantly associated to X count after FDR, including the AG-PG, AG-PIG, anterior inferior isthmus-anterior superior isthmus, and the anterior superior splenium-anterior inferior splenium distances.

The X chromosome count among males was robustly associated with a large number of BLDs after FDR. Because the enumeration of all BLDs that survived FDR would be too extensive, we refer the reader to Figure 5f for a summary of the results. Y chromosome count among males was not as strongly associated with various BLD pairings, with several pairings significant prior to FDR but none surviving FDR Figure 5g.

**Figure 10:**
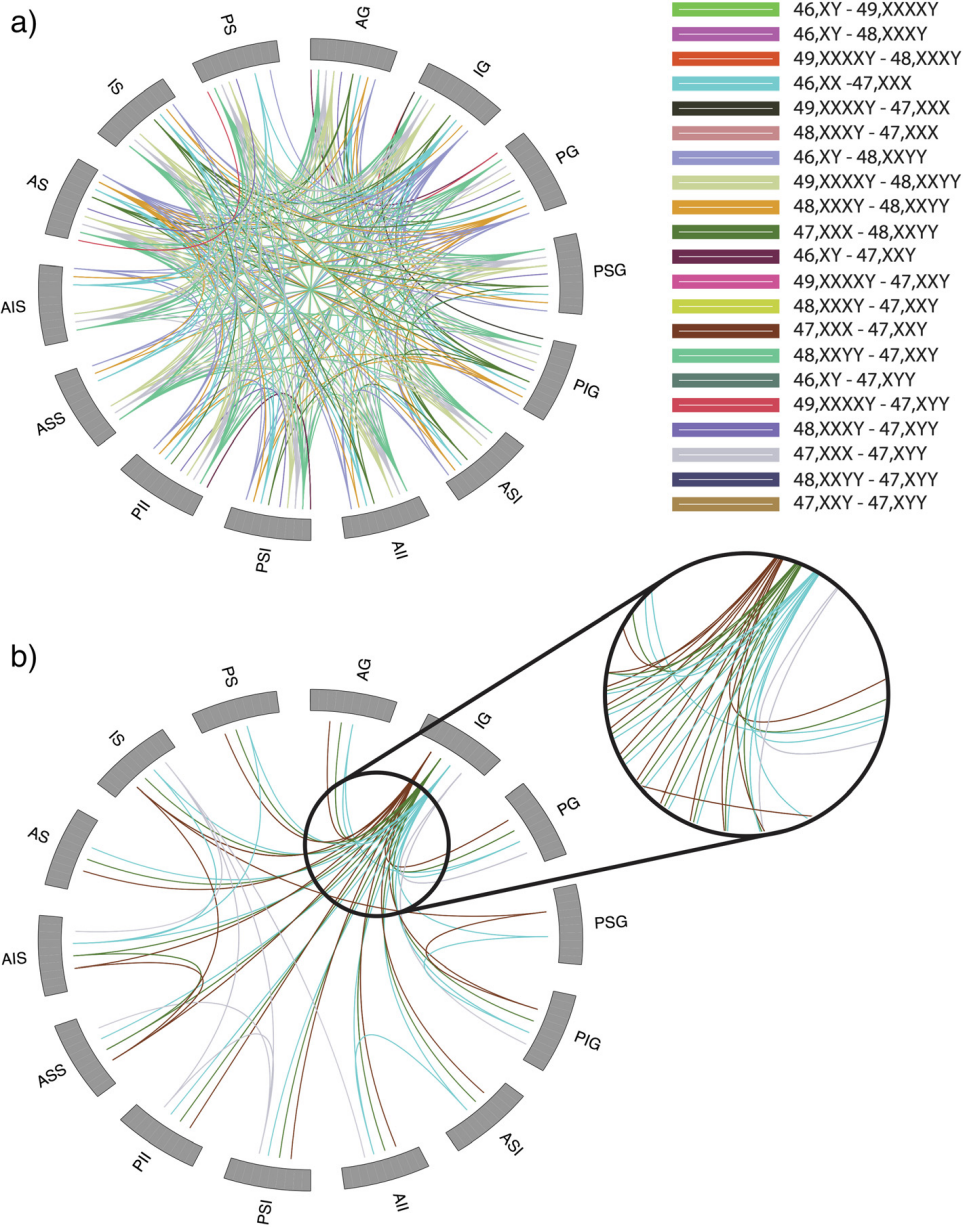
**Circular representation of significant groupwise landmark distances.** Each landmark is represented as a tile on the outer perimeter of the circle. If a between-landmark distance (BLD) is significantly different between two groups then a line color coded with the comparison is drawn between the landmark tiles. **(a)** Significant BLDs prior to multiple comparisons correction. **(b)** Significant BLDs surviving FDR.

An enumeration of the between-group BLDA results would be too lengthy to report. We instead summarize this in Figure 10 which employs a circular plotting model implemented in R version 3.0.2 using an adapted RCircos package [35]. Here, each landmark is represented by a tile along the outer ring of the circles. Where a distance between two landmarks is significant, a line is drawn between the corresponding tiles. Each line is color-coded to correspond to the groupwise comparisons. Figure 10a depicts all significant comparisons prior to correction for multiple comparisons. Figure 10b shows the significant comparisons that survive FDR.

While a large number of pairwise comparisons were significant prior to FDR, a tractably smaller number survived FDR. Eight pairwise groups had significant BLDs passing FDR: (1) 46,XX-47,XXX, (2) 46,XY-48,XXYY, (3) 48,XXYY-47,XXY, (4) 48,XXYY-47,XYY, (5) 46,XY-47,XXY, (6) 47,XXX-47,XXY, (7) 47,XXX-47,XYY, and (8) 47,XXX-48,XXYY. The BLDs remaining significant between 46,XX-47,XXX were highly numerous. The majority of these BLDs are between the IG and other landmarks. The anterior inferior isthmus (AII)-anterior superior isthmus (ASI) distance is also significant between these karyotypes. Among the 46,XY-48,XXYY BLDs, the majority of significant parings exist between the IG and other landmarks as well as the IS and other landmarks. The PG-IG and PIG-IG distances are significantly different between the 48,XXYY-47,XXY karyotypes. The IG-PIG distance is significantly different between 48,XXYY-47,XXY while the IS-IG distance is significant between 46,XY-47,XXY. The comparison of 47,XXX-47,XXY revealed numerous differences between the IG and various other landmarks. The AID-anterior superior splenium (ASS) distance was also significantly different here. The 47,XXX-47,XYY comparison revealed more varied differences. Several distances stemming from the IG were significantly different. Additionally, the PIG-posterior superior genu (PSG), anterior inferior splenium (AIS)-ASS and IS-ASS distances were significantly different between the groups. Significant differences between 47,XXX and 48,XXYY consisted of the PG-AG, PIG-PSG, IS-AIS, and PS-AIS distances.

## 4 Discussion

The main finding of this study is the association of callosal morphometry with sexwise dosages of X and Y chromosomes. We have secondarily observed the effects of the ratio of Y:X chromosomes along a proposed spectrum of supernumerary sex chromosome karyotypes. In addition to detecting morphological changes related to chromosome dosages, we have also identified numerous between-group morphological differences in both the area and shape domains related to karyotype. To our knowledge, this study is the first to apply such an in-depth battery of morphological descriptors to the CCs of this rare set of subjects. The findings have important bearings on neurological development and chromosomally driven sexual dimorphisms.

With respect to area analyses, we observed modest associations at the subregional level with sex chromosome counts and the proposed Y:X ratio. Global area was only found to be related to X counts in the female-only groups. Groupwise comparisons of local and global area demonstrated differential effects of chromosome dosages on the CC regions. The most notable effect appeared in CC region 2 which corresponds to WM paths of the premotor, supplementary motor, and primary motor cortices. Interestingly, a number of cases have reported motor impairments in SCA subjects. The observed reductions we report here may reflect underlying deficits in the motor functions of these subjects [36]–[39]. As androgens may have a protective effect on motor neurons [40], the presence of supernumerary X chromosomes and the resultant decrease in androgen levels may adversely affect either the development or long-term health of motor neurons. In line with this, Ross et al. [41] noted significant motor deficits in boys with Klinefelter’s syndrome (47,XXY). Specifically, 47,XXY boys tested lower in levels of speed, strength, and agility. Salbenblatt et al. [42] reported similar deficits in both gross and fine motor control in 47,XXY boys. Taken together, our findings may provide a neurological basis for these findings.

In addition to the androgen deficits sSCA_X_ males present, they also suffer from hyperestrogenism which is thought to similarly contribute to their social and cognitive impairments [43]. Estrogen is well documented to affect learning, neurological development, and mood [44],[45] in women. However, estrogen has been shown to affect male cognition as well [46],[47] through the conversion of testosterone to estrogen by brain aromatization [48]. As highlighted by recent reports, estrogen has differential effects on male and female cognition and physiology [49],[50]. In females, estrogen levels have been linked to neuroprotective properties such as stroke recovery and Alzheimer’s disease resilience [44]. Little is established about the role of hyperestrogenism in sSCA_x_ males as it is often challenging to disentangle the effects of aberrant sex hormone levels from other concomitant genetic abnormalities. Whether estrogen confers additional neuroprotective properties to these subjects is unknown, however, it is doubtful.

BLDA complemented the raw area analyses, revealing local karyotype-specific expansions or contractions of the CC. Landmarks reflecting the bulbosity of the CC’s rostrum were most correlated with the Y:X spectrum. However, these findings were not robust enough to survive FDR. The X chromosome count among females was robustly associated with the bulbosity of the genual region as well as midline thickness. The significance of the PS-ASS landmark in females also suggests that the length of the splenium is also reduced in 47,XXX relative to 46,XX.

Among the males, we were able to detect BLDs associated with both the X and Y chromosome counts. We were able to see widespread effects of X chromosome dosage among males, and BLDA highlighted many of these effects. We identified a robust association of the AG-PS distance with X count, indicating a lengthening of the CC with additional X’s. The significance of the AII-ASI and posterior inferior isthmus (PII)-posterior superior isthmus (PSI) landmark pairings among male X counts indicates a significant reduction of the mid-body of the CC with increasing Xs. The absence of significant BLDs among the AG-IG-PG-PSG landmarks suggests that the bulbosity of the genu is not strongly affected by X count in males. Similarly, the landmarks associated with the bulbosity of the splenium (AS-PS-IS) appear relatively unrelated to X count. While the significance of associations between BLDs and Y counts did not survive FDR, clear trend-level effects were present. Principally, the BLDs related to Y counts indicated local expansions of the CC, as was expected. The trend of the PG-AG BLD was to increase with additional Ys, indicating an expansion of the genu-rostrum area. Similarly, the IS-AIS distance tended towards elongation with added Ys suggesting an increased length of the splenium. However, the pre-FDR significance of the PII-PSI BLD indicates a shrinkage of the posterior mid-body of the CC. Taken together, the findings of X and Y count among males seems to corroborate the original hypothesis of Y-based expansion and X-based contraction of CC morphometry.

We performed groupwise BLDA to identify specific differences in landmark morphology between the sSCA groups. The most prevalent differences existed between the 46,XX and 47,XXX groups. Nearly all differences stemmed from distances between the IG and a variety of other landmarks. In 46,XX, the distances between the IG and posterior body landmarks was greater than those in 47,XXX suggesting a possible lengthening of the CC in controls. Between the 48,XXYY and 46,XY groups, all significant BLDs indicate an expansion of the 48,XXYY group relative to controls. Similarly, the comparisons between 48,XXYY and both 47,XXY and 47,XYY indicate an expansion of the genu’s bulbosity in the 48,XXYY group relative to these other sSCA karyotypes. The 46,XY-47,XXY comparison reveals an expansion in the distance between the inferior genu and the inferior splenium in the 47,XXY group relative to controls. However, the overall length of 47,XXY, as given by the AG-PS distance, was not significantly greater, suggesting that the effect may reflect a difference in the rostrum of the 47,XXY group. Distances between the 47,XXX and 47,XXY group consisted primarily of distances paired with the IG being larger in 47,XXY than in 47,XXX. The AIS-ASS distance was also larger in 47,XXY indicating a relatively thicker posterior body of the CC in 47,XXY. Between 47,XXX and 47,XYY, the distances between the IG and AG and PG and PIG are all larger in the 47,XXX group suggesting an increased bulbosity of the genu in this group relative to 47,XYY. The AIS-ASS distance is larger in 47,XYY suggesting a thicker posterior body of the CC. The larger distance between the IG and PS in the 47,XYY group suggests an overall longer CC length in the male group. Among the 47,XXX and 48,XXYY groups, the PG-AG and PIG-PSG distances are larger in 48,XXYY suggesting an increased genual bulbosity of the CC. The IS- and PS-AIS distances are also larger in the 48,XXYY group relative to the 47,XXX group suggesting a lengthening of the splenium in the male sSCA group.

The local curvature of the lower boundary of the CC (*κ*_*l*_) was most widely predicted by X count in both males and females prior to FDR. However, these associations failed to survive correction for multiple comparisons. The Y:X karyotype spectrum and raw Y count similarly failed to predict lower boundary curvature profiles after FDR. However, if we consider these as merely trend-level effects, it appears that X count does have a widespread affect on the curvature of the lower CC. As suggested previously, this may be due to ventricular expansion in sSCA_x_ karyotypes more readily altering the shape of the lower, rather than the upper, CC. The effects of chromosome dosage was less widespread in the upper boundary curvature (*κ*_*u*_). However, two associations managed to survive FDR: increased X count appears to lessen *κ*_*u*,1_ in males while an increased Y:X ratio is associated with higher degrees of *κ*_*u*,3_. The groupwise comparisons between curvature profiles do not reveal consistent patterns and is more difficult to interpret at a functional level. However, this highlights the degree of variation among these sSCA groups while providing a valuable morphometric characterization of the spectrum of karyotypes.

Our study has some limitations that merit discussion. Primarily, our sample sizes for large supernumerary karyotypes, while large relative to other studies, are still small. This is an inherent limitation of studying disorders with low prevalence. The small sizes of these groups, specifically 48,XXXY and 49,XXXXY (*N* =5 and 6, respectively), necessarily limits our power to make inference. We used non-parametric resampling techniques to avoid assumptions of sample normality required by parametric tests, but even resampling does not circumvent the issue of having small sample sizes. Some studies address this issue by pooling similar diagnostic groups to boost statistical power. We decided to maintain separate groups, however, as our subjects were non-mosaic and these karyotypes exhibit distinct cognitive and somatic phenotypic profiles [51]. Large supernumerary karyotypes are very rare, so we thought it more valuable to explore their characteristics separately.

A second limitation is the use of a low-pass Gaussian kernel to smooth the callosal boundary. In the process of smoothing, callosal rostrums that are thin and highly hooked are rounded off. The fibers of the rostrum are responsible for connecting the orbital cortices, which may be affected in SCA disorders. However, when the CC is extracted in its binary representation, its boundary is somewhat jagged. It would be favorable to work with a smooth boundary especially when investigating curvature, which is a second-degree derivative and, therefore, highly perturbed by sudden changes or noise. We experimented with a wide range of kernel parameterizations, observing the tradeoff in smoothness to rostrum loss and arrived at our present parameterization as best.

Our results agree with the literature on MRI studies in SCA subjects. These reports primarily focus on 47,XXY [12]–[14],[52]–[54] and, to a lesser extent, higher order supernumerary X karyotypes [22],[55],[56]. These studies consistently report overall reductions in WM/GM volumes and higher ventricular volume. A few studies have also investigated the CC in these groups and indicated a general trend of lower area, which is consistent with a decreased WM volume as well as our present results. Far fewer studies have addressed the phenotypes associated with supernumerary Y subjects, and the reports have often been inconsistent. Some studies suggest a higher ICV in sSCA_Y_[57], but others report little or no change [12]. In our 47,XYY sample, ICV, WM, and GM volumes were all significantly larger than 46,XY.

Using BLDA, we observed a significant inverse correlation of CC thickness with the karyotype spectrum. In fact, many subjects with large *sSCA*_*X*_ karyotypes have noticeably thinner CCs upon visual inspection. In extreme cases, partial agenesis of the CC may occur in these groups [22]. The bulbosity of the splenium and genu were also moderately associated with the chromosome dosages. Because impaired executive function has been reported across sSCA groups [1],[10],[11],[58], the observed reduction in the genu’s bulbosity with increased dosages makes sense as these WM paths connect the frontal cortices. However, this effect was only present using BLDA and not simply area-based analysis.

To our knowledge, this is the first study to simultaneously investigate this set of sSCA karyotypes, not only in relation to each other and matched controls but also along a dosage-ratio spectrum and by looking at X and Y counts within sex. Our use of several morphological descriptors is also novel. While analysis of area is intuitive, shape-based metrics further inform us about morphological profiles for various diagnostic groups.

## 5 Conclusions

Our results reinforce several prior findings about the pattern of CC area reduction in *sSCA*_*x*_ while also introducing several new findings. The size of primary and secondary motor CC regions appears to be affected by X dosage. Additionally, the bulbosity of regions associated with frontal WM seems strongly linked to X dosage. These findings encourage us to explore whether chromosome dosage has an effect on WM tractography in other brain regions. Future studies involving DTI and shape analysis of other subcortical structures in these karyotypes will be beneficial.

## 6 Endnotes

^a^A failure of homologous chromosomes to separate after metaphase.

^b^In cytogenetic nomenclature, the comma separates the total chromosome number from the sex chromosomes.

^c^A condition in which different cells of the same individual express more than one karyotype.

^d^WM hyperintensities often reflect underlying lesions or other varied pathologies of the WM fibers.

^e^*q* values are FDR-adjusted *p* values.

## Abbreviations

sSCA: supernumerary sex chromosome aneuploidies

s*SCA*_*Y*_: Y-variant sSCA

*sSCA*_*X*_: X-variant sSCA

BLDA: between-landmark distance analysis

*K*: curvature

*K*_*l*_: lower boundary curvature

*K*_*u*_: upper boundary curvature

EDMA: Euclidean distance matrix analysis

AG: anterior genu

IG: inferior genu

PG: posterior genu

PSG: posterior superior genu

PIG: posterior inferior genu

ASI: anterior superior isthmus

AII: anterior inferior isthmus

PSI: posterior superior isthmus

PII: posterior inferior isthmus

ASS: anterior superior splenium

AIS: anterior inferior splenium

AS: anterior splenium

IS: inferior splenium

PS: posterior splenium

## Competing interests

The authors declare that they have no competing interests.

## Authors’ contributions

BW designed the study, performed statistical analyses, and wrote the manuscript. SJ designed the method of spatial registration used in the shape analyses, aided in interpretation of results, and assisted in writing the manuscript. MR contributed to interpretation of the results and design of the analyses. JB recruited the participants and acquired neuropsychological data for the study. AT and PT advised the study design, edited the manuscript, and helped interpret the results. JG helped design the study, acquired the MRI images, and helped write and edit the manuscript. All authors read and approved the final manuscript.
